# Deep feature selection using local search embedded social ski-driver optimization algorithm for breast cancer detection in mammograms

**DOI:** 10.1007/s00521-022-07895-x

**Published:** 2022-11-05

**Authors:** Payel Pramanik, Souradeep Mukhopadhyay, Seyedali Mirjalili, Ram Sarkar

**Affiliations:** 1 Department of Computer Science and Engineering, Jadavpur University, Kolkata, India; 2 Centre for Artificial Intelligence Research and Optimisation, Torrens University Australia, Brisbane, QLD 4006 Australia; 3 Yonsei Frontier Lab, Yonsei University, Seoul, South Korea; 4 University Research and Innovation Center, Óbuda University, Budapest, 1034 Hungary

**Keywords:** Mammogram images, Breast cancer, Deep learning, Social ski-driver, Optimization, Local search, Algorithm

## Abstract

Breast cancer has become a common malignancy in women. However, early detection and identification of this disease can save many lives. As computer-aided detection helps radiologists in detecting abnormalities efficiently, researchers across the world are striving to develop reliable models to deal with. One of the common approaches to identifying breast cancer is through breast mammograms. However, the identification of malignant breasts from mass lesions is a challenging research problem. In the current work, we propose a method for the classification of breast mass using mammograms which consists of two main stages. At first, we extract deep features from the input mammograms using the well-known VGG16 model while incorporating an attention mechanism into this model. Next, we apply a meta-heuristic called Social Ski-Driver (SSD) algorithm embedded with Adaptive Beta Hill Climbing based local search to obtain an optimal features subset. The optimal features subset is fed to the K-nearest neighbors (KNN) classifier for the classification. The proposed model is demonstrated to be very useful for identifying and differentiating malignant and healthy breasts successfully. For experimentation, we evaluate our model on the digital database for screening mammography (DDSM) database and achieve 96.07% accuracy using only 25% of features extracted by the attention-aided VGG16 model. The Python code of our research work is publicly available at: https://github.com/Ppayel/BreastLocalSearchSSD.

## Introduction

Breast cancer is a disorder where the cells of the breast tissue alter and divide uncontrollably, resulting in a lump or mass in the mammary glands or between the mammary glands and the nipple in the majority of instances. Unfortunately, breast cancer is one of the most frequent malignancies among women, and it has a high fatality rate. Early identification of breast cancer can significantly improve women’s survival rates, which is critical because breast cancer can be cured in 95 percent of cases if caught early [1]. Reviewing prior diagnostic data and gathering relevant information from past data are key to identifying this disease at an early stage.

Medical images are one of the most important sources of information for the identification and diagnosis of various illnesses and anomalies, allowing radiologists to examine the interior structure of human bodies. It is critical in the diagnosis of clinical diseases, the evaluation of treatment, and the detection of anomalies in various bodily organs such as the eyes [2], lungs [2], brain, breast [3, 4], and stomach [5]. One of the most efficient ways to diagnose breast cancer is through medical imaging. The research in this field has grown significantly over the last three decades. Breast mammography is the most economical among various other detection methods, namely Thermal imaging, Magnetic Resonance Imaging (MRI), Ultrasound imaging, Computerized Tomography (CT), and Histology imaging [6].

A breast mammogram is an X-ray image of the woman’s breast which is a common diagnostic measure for screening for breast cancer. It is useful for the detection of breast swellings, masses, calcifications, and dimpling of breast tissue. All of these are indicating an early stage of breast cancer. However, it is not an easy task to identify these symptoms from the breast images. Moreover, incorrect assessment of these images leads to an incorrect diagnosis with dangerous consequences. Consider the circumstance of a false negative diagnosis, in which an early stage of breast cancer is misdiagnosed as a normal case. As a result, the individual’s chances of surviving five years are reduced [3].

Over the last decades, researchers have leveraged various machine learning (ML) techniques in the medical image analysis domain to help with decision-making processes. Data analysis, data cleaning, and meaningful feature extraction or feature representations are the reasons for ML’s success to accomplish several tasks. Medical experts are capable of using their knowledge to relate features of a dataset to real-world phenomena or a fact, which is a challenging task for ML techniques. Deep learning (DL) alleviates this drawback as future engineering and processing is a part of the learning process as opposed to traditional methods with manual processes [7].

Many researchers have exploited DL methods in various domain applications such as in image classification [8], image segmentation [9], security [10–12], reinforcement learning [13], letter Recognition [14], partial differential equation solving [15]. In recent times researchers have successfully explored various DL-based methods in the domain of medical imaging, particularly in disease detection like Alzheimer’s detection [16], fracture detection [17], COVID-19 detection [18], and many more. Generally, DL models require an ample amount of data for proper training purposes and the accessibility of such enormous volumes of data in the medical realm is quite unusual. As a consequence, experts are increasingly embracing the approach of transfer learning, in which Convolutional Neural Network (CNN) models are trained on bigger datasets such as the ImageNet dataset and then the weights are transferred and fine-tuned on a smaller (i.e., target) dataset [19]. Although transfer learning addresses a lot of challenges faced earlier by the researchers, to improve the performance of the models, several standard machine learning methodologies such as feature selection (FS) are now combined with the DL model.

FS is one of the conventional ways to reduce computational efforts that remove redundant features and selects a subset of distinct features. Also due to the presence of redundant features, distinctive features may not be given the importance they should be for classification purposes [20]. In the past, several optimization algorithms based FS methods have been exhaustively exploited in various domains like image enhancement [21], traveling Salesman problem solving [22], security [23], classification [24], SVM parameter optimization [25], and solving class imbalance problem [26]. Moreover, researchers have effectively been able to solve many image classification problems in the medical image analysis domain using this method such as in prostate cancer detection [27], Alzheimer’s disease detection [28], and many more. This encourages us to employ an FS approach that is based on an optimization algorithm in our work.

The concept of embedding a local search method with FS is not very old. Researchers in the recent past have successfully developed and deployed such models and in turn, also proved that these models perform better in terms of classification results [29]. Choosing the optimal feature subset in FS is usually difficult, especially in wrapper-based techniques where the chosen subsets must be assessed using a learning algorithm at each iteration. Ensemble of local search improves an FS method’s exploitation ability and hence improves the overall learning model’s performance.

**Fig. 1:**
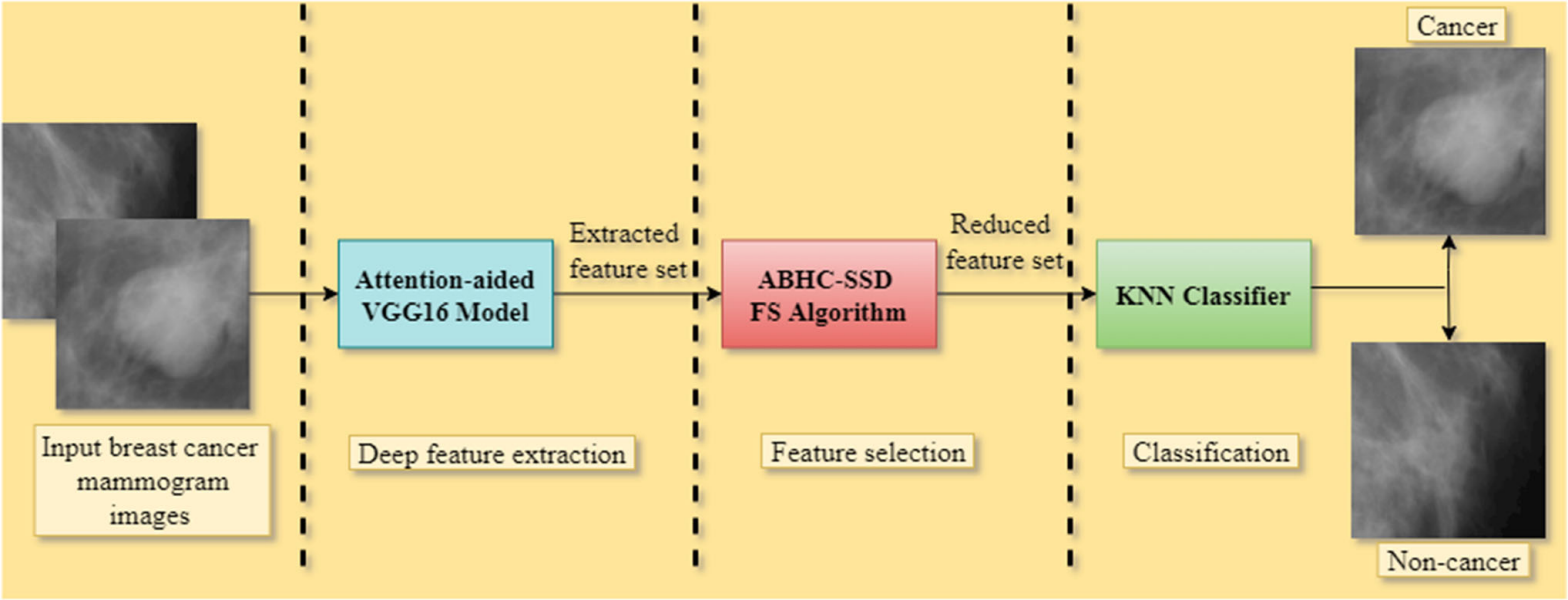
The pipeline of our suggested breast cancer classification model

Because of the above-mentioned facts, in the current work, we design a two-stage breast cancer classification model taking mammograms as input. At first, we use an attention-aided DL model to extract features from the mammograms. Then, we apply a local search embedded FS approach to reduce the feature dimension and augment the classification ability. As the basis model, we used a transfer-learning model that was pre-trained on the ImageNet dataset and fine-tuned it on the target dataset. Furthermore, we introduce attention by incorporating a global weighted average pooling mechanism on the base model. We extract features using this attention-based transfer learning model for the target dataset and lastly process these extracted features with the local search embedded FS method to produce optimal and reduced feature subsets. Finally, these reduced features are used as the inputs to the KNN classifier to produce the final classification results. After experimenting with different transfer learning models and different FS algorithms, we find that the attention-aided VGG16 model and Adaptive Beta Hill Climbing (ABHC) embedded SSD based FS algorithm on the mentioned dataset outperforms other contemporary methods. Figure 1 depicts the whole architecture of the suggested model.

### Motivation and contributions

In the domains of computer vision and image processing for health and medical assessment, integrating DL approaches with FS algorithms has yielded significant results [29–32]. Researchers have shown the immense potential of DL-based applications for mammogram image processing in terms of providing reliable breast cancer predictions [4, 33–35]. Moreover, the attention mechanism exploits the most important regions of an image by paying more attention to the same [36, 37]. Furthermore, FS approaches reduce the number of features, whereas local search helps to increase the exploitation capability of the FS method and produces the most optimal subset of features [29]. These efforts prompted us to investigate how DL methods perform when paired with FS approaches, as well as to confirm the usefulness of the attention mechanism and the capabilities of local search strategies for breast cancer analysis and assessment from mammography imaging. Therefore, in this paper, we have proposed a model in which an intelligent amalgamation of a DL model with an optimization algorithm-based FS approach has been made. The main contributions of the present research work are listed below.

1. We create a model for breast cancer classification from mammograms that combines the principles of deep learning and optimization algorithms.
2. We introduce an attention mechanism on a deep CNN-based transfer learning model, called VGG16, and fine-tune it for the extraction of deep features from the input images.
3. We ensemble a local search, namely ABHC with SSD based FS algorithm to produce an optimal feature subset from the features produced by the CNN model.
4. We achieve state-of-the-art classification accuracy with just 25% of features of the original feature set obtained by the CNN model when evaluated on the DDSM database.

The rest of this research study is broken down into categories. Section 2 is a literature review in which we look at several techniques of breast cancer diagnosis, starting with mammography and then moving on to local search-based FS algorithms. Section 3 lays out the requirements for this research project, followed by a detailed description of our proposed study in Sect. 4. The metrics we employ to evaluate the proposed model and analyze the experimental results are then discussed in Sect. 5. Finally, in Sect. 6, we make some concluding remarks and discuss some potential future directions.

## Literature survey

This section contains two subsections, wherein in the first subsection we discuss some recent DL-based methods for the detection and classification of breast cancer, and in the subsequent subsection, we discuss the application of FS algorithms in the medical domain, specifically, in breast cancer detection.

### DL-based methods for breast cancer detection

In health care systems, there is various machine and DL approaches developed by researchers. Machine learning is widely utilized in several domains like health care, early disease detection, biomedical, etc. [38]. In recent times, the advancement in machine learning, especially in DL has created a significant impact on the medical imaging field. It enhances the precision to identify, classify, and quantify patterns in medical images. To be precise, exploiting complex representations of the features which are understood or realized from the input data is the prime reason for this tremendous progress. Thus, DL models are achieving extraordinary results in different medical applications [7].

For instance, A. Saber et al. have shown in [33] a DL model based on the transfer learning technique for the detection of breast cancer from the mammographic image analysis society (MIAS) database. In this, the authors showed some pre-processing techniques and discussed the evaluation metric results of different pre-trained CNNs for the MIAS database. The experimental results showed that out of different pre-trained CNNs, the VGG16 model yields the best result, which is seen in our research work as well. Similar work can be found in [39]. In 2019, Shen et al. [8] proposed a CNN model to classify mammogram images of the CBIS DDSM dataset. This method yields an AUC score of 0.88, sensitivity of 86.1%, and specificity of 80.1% which is quite low. Furthermore, the INBreast dataset was used to increase the performance of the model yielding a 0.95% AUC score, 86.7% sensitivity, and 96.1% specificity. In the next year, Khatami et al. [40] introduced a regularization scheme for the detection of cancer from mammograms by changing the weights of the convolutional layers using some additive noise and achieved an accuracy of 83%. The limitation of this model is the lower detection accuracy which may not be useful in practical scenarios. A paper published in 2015 by Ertosun and Rubin [34] used a DL-based method which achieves 85% accuracy for identifying breast images with a mass from breast mammograms and an accuracy of 85% for mass localization in mammograms with an average false positive rate of 0.9 per sample image. However, their main focus is to find out whether the breast mass is benign or malignant. In another work, Levy et al. [4] have come up with a DL model with pre-processing and data augmentation to classify pre-detected breast masses from DDSM mammograms and achieved 92.9% accuracy, whereas Khamparia et al. [3] have proposed a method using the fine-tuned VGG16 transfer learning model to diagnose breast cancer from DDSM mammograms, and achieved an accuracy of 88.3%. The authors have used data augmentation and regularization to enhance the performance of the model. A study has been done by L. G. Falconi et al. [41] of different transfer learning models like NasNet, MobileNet, VGG16, ResNet, Xception, and Resnext to train a breast abnormality malignancy classifier. In this study, the authors have concluded that training of DL models tends to overfit and fine-tuning of the models achieves a better classification performance in the case of the VGG16 model which gives an accuracy of 84.4% in the CBIS-DDSM dataset. Al-antari et al. [35] have proposed a CAD system based on You Only Look Once (YOLO) to detect and classify breast lesions. In this work, the authors used the YOLO detector to detect breast lesions from the DDSM and the InBreast mammograms, and the classification was done using three DL classifiers, namely regular feed-forward CNN, ResNet-50, andInceptionResNet-V2. From these research works, it can be said that automatic DL models can achieve better results even on heterogeneous mammography platforms. Also, it holds a strong promise for improving the performance of the clinical tools for reducing false positive and false negative screening mammography results.

Researchers have successfully explored the utilization of ML and DL models not only on mammograms but also on different breast cancer image modalities available. For instance, In Vahadane et al. [42], authors have introduced a structure-preserved stain-normalization technique to deal with histopathological images and achieved 87.50% classification accuracy. In another work, Sarmiento et al. [43] proposed a machine learning-based technique for automatic breast cancer grading of histological images in which the extracted feature from various characteristics of the image such as texture, color, and shape was fed to the Support vector machine (SVM) classifier as the input and with tenfold cross-validation, this method achieved an accuracy of 79.2%. In another research paper by Nawaz et al. [44], the authors used a fine-tuned AlexNet for breast cancer classification in histology images and achieved an image-wise accuracy of 75.73% and patch-wise accuracy of 81.25%. In [45], Silva et al. have suggested a method for abnormality detection in breast thermal images. The authors used Auto-WEKA with some defined settings for the selection of best features and used a K-star classifier with a tenfold cross-validation method for the classification of images. Also, in [46], the authors have introduced a deep CNN method for the automatic cancer tissue nuclei detection, segmentation, and classification of breast cancer cells from whole slide images of hematoxylin and eosin stains. In this work, a multilevel saliency nuclei detection model is used for the detection of nuclei, and the same is integrated with the deep CNN model for the classification of benign and malignant cells. Rakhlin et al. [47] have designed a DL model to classify the images of breast tissues. In this work, pre-trained models of VGG-16, InceptionV3, and ResNet-50 are used for feature extraction, whereas for the classification purpose a tenfold cross-validation scheme with Light Gradient Boosting (GBM) classifier has been used. This approach achieves an accuracy of 87.2% for breast cancer image classification.

However, it can be seen that researchers have extensively used DL models as well as transfer learning models for breast cancer detection from different medical imaging modalities available. But considering breast cancer detection, it is important to focus on the region of interest (ROI) as some of the regions may be more relevant than others, thus justifying the need of adding an attention mechanism to a DL model. In this work, we try to explore the attention mechanism of transfer learning models for breast cancer classification from mammography.

### Nature-inspired meta-heuristic FS algorithms for breast cancer detection

Nature-inspired meta-heuristic techniques and their variants are widely used in solving FS problems [48]. The field of meta-heuristics is vast and it has made significant advancements toward solving complex optimization problems. Since the first meta-heuristic was presented, a considerable amount of progress has been achieved, and countless new algorithms are offered regularly. There is no dispute that research in this area will advance in the coming future. There are two primary groups of meta-heuristic algorithms. One is meta-heuristic algorithms based on a single solution, where optimization begins with a single solution and gets updated as the algorithms move through iterations. Another group is population-based meta-heuristic algorithms, where optimizations start with a population of solutions and update it over the iterations. However, the first group of algorithms can get trapped within local optima and they only partially explore the search space, whereas the latter group helps to prevent local optima since they have a great search space exploration opportunity and various solutions that work together to help one another. Meta-heuristic algorithms can be classified based on their behavior into four categories: algorithms based on physics, swarm intelligence, evolution, and humans [90–93].

Meta-heuristic techniques yield an optimal solution by iteratively exploring as well as exploiting the search space. It assists to select an optimal set of features so that a better classification performance will be ensured with that set of features. Every meta-heuristic method tries to maintain a good balance between exploration and exploitation of the search space to improve the results [49]. Researchers have effectively been able to solve many image classification problems in the cancer detection domain as well as other medical domains [49–51] using several meta-heuristic-based FS algorithms.

For instance, in 2010, Gandhi et al. [52] suggested a cancer detection method based on Pittsburgh Learnt Fuzzy Rule and Particle Swarm Optimization. In 2014, Ahmad et al. [53] designed a breast cancer diagnosis model by using the Genetic Algorithm (GA) for both FS and parameter optimization of an artificial neural network (ANN). The higher computational cost was the main drawback of both these methods. In 2019, Huang et al. [54] proposed a new breast cancer diagnosing technique based on the fruit fly optimization algorithm embedded with a Levy flight strategy. It was mainly used to optimize two key parameters of the SVM classifier. They have exploited two datasets—Wisconsin Prognostic Breast Cancer dataset and Wisconsin Diagnostic Breast Cancer dataset for result computation. In the same year, Sayed et al. [55] suggested a hybrid model on the same dataset that exploited cluster analysis algorithm and binary version of Moth-flame optimization and Whale optimization algorithm (WOA) for FS. In 2020, Fang et al. [56] proposed an intelligent amalgamation of multi-layer perceptrons with WOA for breast cancer detection. Lower accuracy was the main limitation of these techniques. In 2021, Oyelade et al. [57] suggested a nature-inspired meta-heuristic optimized convolutional neural networks model detect abnormalities in breast cancer images. It involved training a CNN network using GA, WOA, multiverse optimizer (MVO), satin bower optimization (SBO), and life choice-based optimization (LCBO) algorithms to optimize only the weights and bias of the model. The main drawback of this model was lower accuracy. In the same year, Tavasli et al. [58] proposed an ensemble with a soft-weighted gene selection-based model for the classification of cancer using an improved version of the Water Cycle Algorithm. This model lacked generalizability and accuracy. Also, Rezaee et al. [59] have suggested a model for identifying multi-mass breast cancer following hybrid descriptors and memetic meta-heuristic learning. Drawbacks were large data processing time and lower precision.

To the best of our knowledge, the SSD optimization algorithm has not been used yet in the domain of breast cancer detection. In this work, we explore this novel optimization algorithm for FS and achieve promising results. However, research works with SSD in the medical domain can be found in [60–62].

## Preliminaries and essential definitions

In this section, we mention some prerequisites which are needed to describe and understand our proposed model. We briefly discuss the VGG16 model, used for feature extraction from the input images, and SSD and ABHC algorithms that are collectively used to eliminate the irrelevant features obtained in the previous stage.

### VGG16

**Fig. 2:**
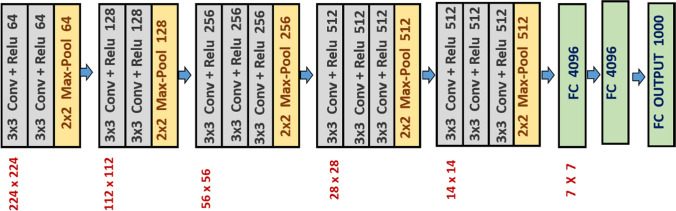
Detailed architecture of the VGG16 model including fully connected layer and 1000-dimensional output layer for the ImageNet database

VGG16 network is proposed by K. Simonyan and A. Zisserman [63]. It is a very basic CNN model having 13 convolutional layers of 3 × 3 filters with a stride 1, 5 max-pooling layers of $2\times 2$ filters with stride 2, and 2 fully connected layers (FC) followed by a softmax for the output layer (Fig. 2). The model obtained a test accuracy of 92.7% (top-5) on the popular large-scale ImageNet dataset [63]. This network has approximately 138 million parameters and 16 in VGG16 refer to that it has 16 weighted layers.

### Social ski-driver optimization algorithm

SSD is a recent optimization technique suggested by Tharwat et al. [25]. It is based on the approach taken by ski drivers when they go downhill. Various modules of SSD are discussed as follows:

#### Location of the agents

The location of the agents ($${L}_{k}^{{R}^{n}})$$ is exploited to compute the fitness function at the particular position of a multi-dimensional search space.

#### Best personal location

At each iteration**,** the fitness measure (described in Sect. 4.3) for every agent gets matched with the personal best fitness measure (previously obtained) and that best location is saved as the personal best location ($${PB}_{k})$$ for that agent.

#### Best mean global location

The agents proceed toward the best global location according to the algorithm. The best global location is computed as the average of the locations of the top 3 solutions as calculated in Eq. 1 and denoted as MGB:

1 $$MGB = \frac{{L_{x} + L_{y} + L_{z} }}{3}$$

$$where\; L_{x} \;,L_{y}\; and \;L_{z } \;are\; the\; locations\; of \;top\; 3 \;solutions\; respectively.$$

#### Velocity and location updating

The location and velocity of the agents are modified according to Eqs. (2) and (3), respectively:

2 $$L_{k}^{T + 1} = V_{k}^{T} + L_{k}^{T}$$

3 $$ V_{k}^{T + 1} = \left\{ {\begin{array}{*{20}c} {h*\sin \left( {rand\left( {0,1} \right)} \right)\left( {PB_{k}^{T} - L_{k}^{T} } \right) + \sin \left( {rand\left( {0,1} \right)} \right)\left( {MGB_{k}^{T} - L_{k}^{T} } \right)} & {if \;rand\left( {0,1} \right) \le 0.5} \\ {h*\cos \left( {rand\left( {0,1} \right)} \right)\left( {PB_{k}^{T} - L_{k}^{T} } \right) + \cos \left( {rand\left( {0,1} \right)} \right)\left( {MGB_{k}^{T} - L_{k}^{T} } \right)} & {else} \\ \end{array} } \right. $$

In Eqs. (2) and (3),$V_{k}^{T} , MGB_{k}^{T} ,L_{k}^{T}$ represent the velocity, mean global best position, and current position of the particle at $k$th dimension and $T$th iteration, respectively. $PB_{k}$ represent the personal best position of the particle at $k$th dimension. $Sin\left(x\right) and Cos(x)$ are traditional sine and cosine functions. $rand\left(\mathrm{0,1}\right)$ function chooses any real number between 0 and 1. $h$ is a variable that is exploited to maintain the parity to achieve a balance among both two crucial elements of exploitation and exploration, and it is computed according to Eq. 4:

4 $$ h^{T + 1} = r \times h^{T} $$

In Eq. (4), $T$ denotes the present iteration and $r$ is exploited to decrease the value of $h$. In Eq. (3), deriving $V_{k}^{T + 1} ,$ the sine and cosine functions guarantees that the directions of movement of the agents are not very straightforward. This is because those functions allow the algorithm to explore and it helps to diversify the searching domain but in a controlled way. Another advantage of the SSD algorithm is that it is comparatively more social than other meta-heuristics. The agents in SSD try to get to the mean of the best three options. As a result, if the global best solution is found to be in local minima, the SSD can use the other two best solutions for escaping [25]. SSD is quicker at discovering optimal solutions than the PSO algorithm.

### Local search (ABHC)

Local search is used as a heuristic method for solving computationally expensive optimization problems. Local search is applicable for the problems that can be framed as searching for a solution maximizing or minimizing a criterion among a huge domain. Local search explores from solution to solution in the search space by doing some local changes, until a chosen solution seems to be optimal or the number of iterations ($MT$) is finished. ABHC [64] is one of the popular local search methods we have used in this work. Hill climbing sometimes faces problems in local optima. To get rid of this problem, ABHC [64] is proposed. This algorithm inputs an agent $L$ location and outputs a modified location of the agent in the search space. This algorithm depends on two operators—one is the Neighborhood operator ($N$) and another one is the $Beta$ operator. Here, $N$ operator randomly chooses a neighbor $L^{^{\prime}} \left( {L_{1}^{^{\prime}} ,L_{2}^{^{\prime}} ,L_{3}^{^{\prime}} \ldots .L_{k}^{^{\prime}} } \right)$ from a solution $L\left( {L_{1} ,L_{2} ,L_{3} \ldots .L_{k} } \right)$ as follows:

5 $$ L_{j}^{^{\prime}} = L_{j} \pm rand\;\left( {0,1} \right) \times N \;\;\;where\; j = 1,2, \ldots k $$

In Eq. 5, $N$ is the greatest probable distance between the present solution and the neighbors, $rand\left(\mathrm{0,1}\right)$ is a function to generate random numbers between 0 and 1. $Beta$ operator gets motivated by the mutation operator used in GA. We assign values to new solutions either arbitrarily from the comparable domain with a probability $Beta$ = $rand\left(\mathrm{0,1}\right)$ or the present solution as follows:

6 $$ L_{j}^{^{\prime\prime}} = \left\{ {\begin{array}{*{20}c} {L_{j} } & {if\; Beta > rand\;\left( {0,1} \right)} \\ {L_{j}^{^{\prime}} } & {otherwise} \\ \end{array} } \right. $$

In Eq. (6), $L_{j}^{^{\prime\prime}} , L_{j}\,\, and\,\, L_{j}^{^{\prime}}$ denote the $j$th dimension of the updated location of the solution, previous solution, and neighborhood of the previous solution, respectively.

Now, the outcome of this version of hill climbing is mostly dependable on the values of $Beta$ and $N$. Determining the values of these two parameters needs comprehensive experiments. To bypass this shortcoming, ABHC came into existence. In ABHC, $Beta$ and $N$ are the functions of the number of iterations.

$N\left( z \right)$ is the functional measure of $N$ in the $z$^th^ iteration. $N\left( z \right)$ can be determined according to Eq. (7).

7 $$N\left( z \right) = 1 - \frac{{z^{\frac{1}{c}} }}{{MT^{\frac{1}{c}} }} \;\;where\; c = cons\tan t$$

Here $MT$ denotes the maximum number of iterations and $z$ is the current iteration number.

The value of $Beta$ in $z$th iteration is denoted as $Beta(z)$ as follows:

8 $$ Beta\left( z \right) = \frac{{\left( {Ma - Mi} \right) \times z}}{MT} + Mi $$

In Eq. (8), $Ma$, $Mi,$ and $z$ are the maximum and minimum values of $Beta$ and the current number of iterations, respectively. Now, if the newly generated neighbor $L^{\prime\prime}$ is better than $L$, then $L$ is replaced with $L^{\prime\prime} .$
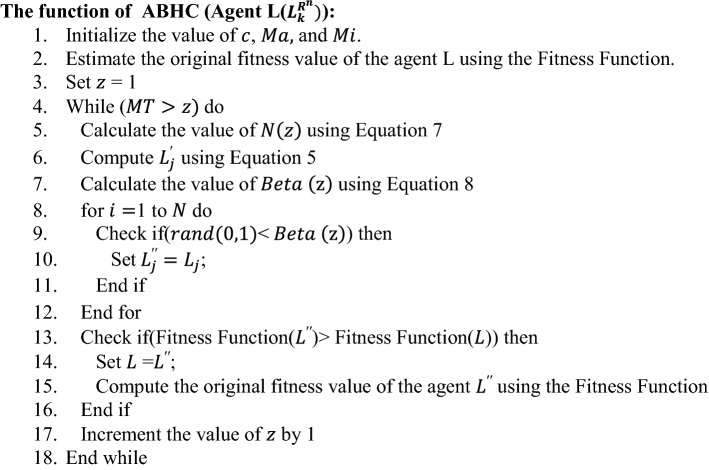


## Proposed model

As previously stated, we create a two-stage breast cancer classification model in this study. To extract features from the mammograms, we first utilize an attention-aided DL model. Then, to minimize the feature dimension and improve classification ability, we use a local search integrated FS technique. We detail the complete process of our work in this part.

### Feature extraction from the attention-aided DL model

For feature extraction from mammography inputs, we employ a deep attention model. We start with the VGG16 model, which has been pre-trained on the popular ImageNet dataset, and add an attention mechanism. We investigate the VGG16 model minus the top layer, which has fully linked layers and freezes the weights of all levels to prevent the layers from learning new information during the model’s training. The Global Average Pooling (GAP) layer takes the role of the fully linked layer.

**Fig. 3:**
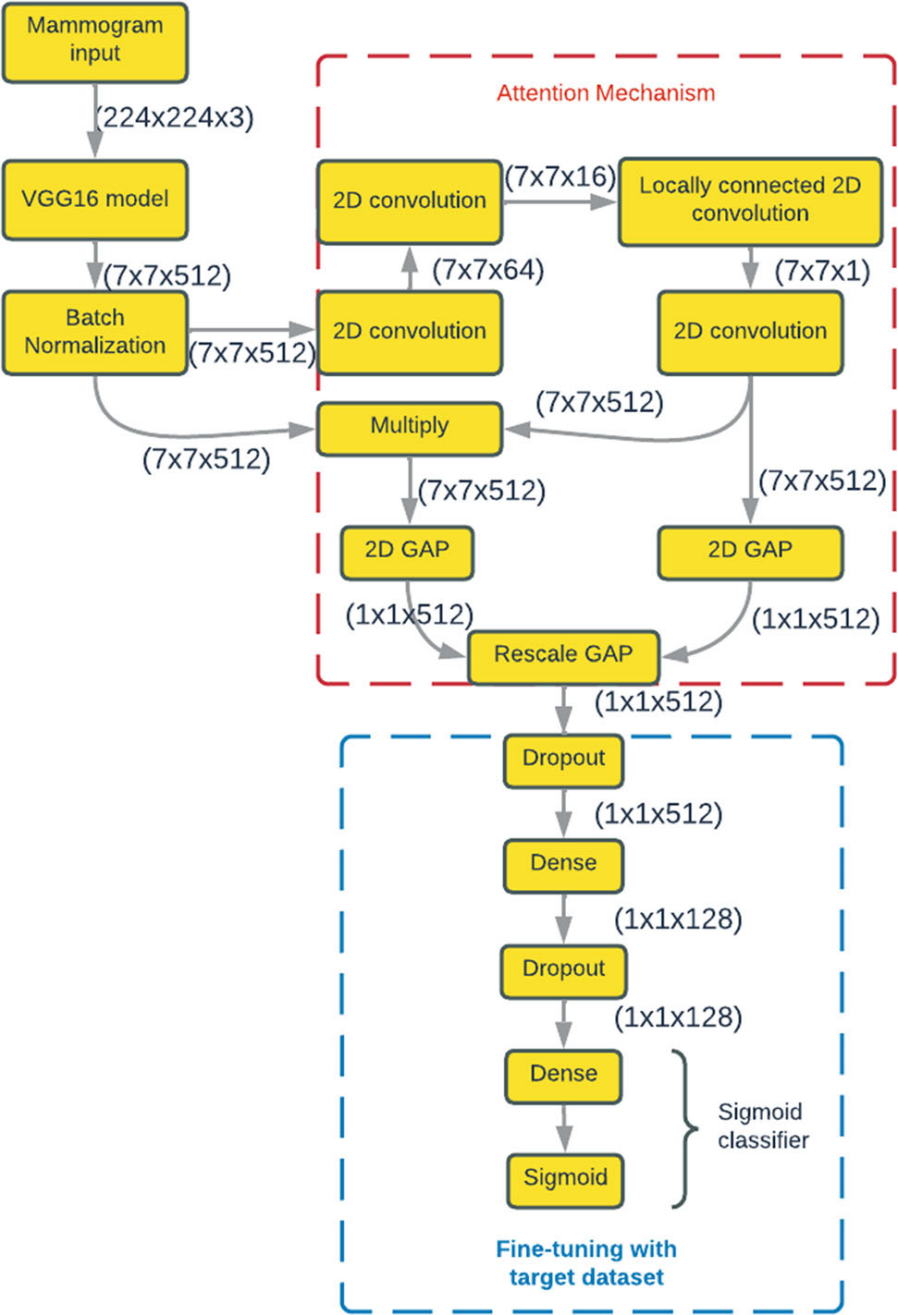
Architecture of the attention-aided VGG16 model with a weighted average GAP layer (all the layers along with input and output shapes). Area of the attention and the fine-tuning of the model are highlighted by red and blue colored dashed boxes, respectively

GAP [65], a pooling operation, is usually applied in place of fully connected layers in classical CNNs. Similar to simple pooling (max-pooling or average pooling) layers, it reduces the spatial dimension of a given tensor. For instance, a three-dimensional tensor having dimensions $\mathrm{h}\times \mathrm{w}\times \mathrm{d}$ gets converted to the dimension of $1\times 1\times d$. GAP produces a single value for each feature map of dimension $h\times d$ by taking the average of all $hw$ values. In this work, in the final convolutional layer of the base VGG16 model instead of adding fully connected layers, the generated vector is supplied to the final layer after we take the average of the feature maps. The GAP layer summarizes the spatial records through imposing correspondences among feature maps and categories, consequently making it robust in terms of spatial translation of the input data. However, only GAP is too simplistic as some regions may be more significant than others, thus needing attention. Here, we introduce an attention method to turn on some pixels in the GAP layer. We add one locally connected convolutional layer of kernel size 1 × 1 and fan it out to all the layers of the model. Next, we add a lambda layer [66] to account for missing values from the attention model that rescale the results based on the number of pixels. Lastly, we fine-tune the model by adding dropouts to prevent over-fitting the model during training [67]. Thus, we propose a weighted GAP to the existing CNN architecture as depicted in Fig. 3.

### FS and classification using local search embedded SSD algorithm

An FS problem seeks to find the best subset of features from the main set to augment the accuracy of a learning model. It can also be considered as a dimension reduction algorithm that removes redundant and/or highly correlated features. Due to the binary nature of this problem, most meta-heuristics are not able to solve them. This is due to the assumption of continuous variables in the vanilla version of such algorithms. There are different methods in the literature to convert them to binary algorithms. One of the most computationally cheap ways is to use a transfer function. In Particle Swarm Optimization (PSO) algorithm where a velocity vector is used to update solutions, this transfer function relates velocity to the probability of changing the position in a binary space. The transfer function [29] used in the current work is shown in Eq. (9).

9 $$V\left( x \right) = \frac{\left| x \right|}{{\sqrt {1 + x^{2} } }}$$

Exploiting the $V$-shaped transformation function the location of an agent is modified as per the below Eq. (10).

10 $$ LB_{j}^{k + 1} = \left\{ {\begin{array}{*{20}c} {c(LB_{j}^{k} )} & {if\; V\left( {LB_{j}^{k + 1} } \right) > rand\left( {0,1} \right)} \\ {LB_{j}^{k + 1} } & {otherwise} \\ \end{array} } \right. $$

**Fig. 4:**
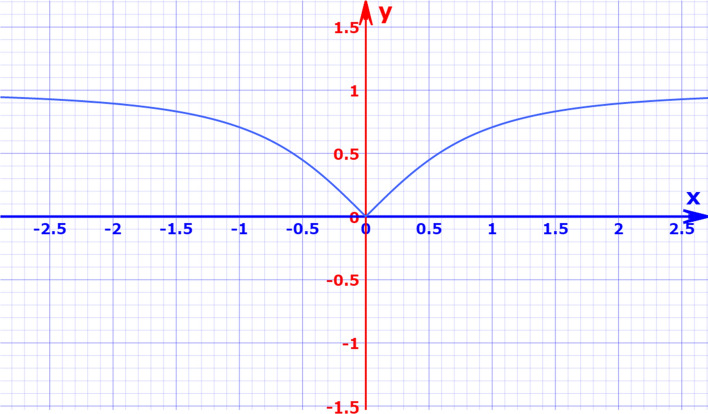
Graphical representation of the V-shaped transfer function


In Eq. (10), $LB_{j}^{k + 1}$ denotes the agent’s modified location, $LB_{j}^{k}$ signifies the location of the agent at that particular time(here $k$ denotes iteration number and $j$ denotes number of dimention) and $rand(\mathrm{0,1})$ is a function that generates random numbers between 0 and 1. The function $c(x)$ denotes the complement function for all binary x i.e.,  $c\left(x\right)=1-x$. Figure 4 shows the graph of the V-shaped transfer function. After altering an agent’s location in each iteration, ABHC is used to optimize the position of the agents to get a higher fitness value. The SSD algorithm’s exploitation potential is improved by using an ABHC-based local search technique.

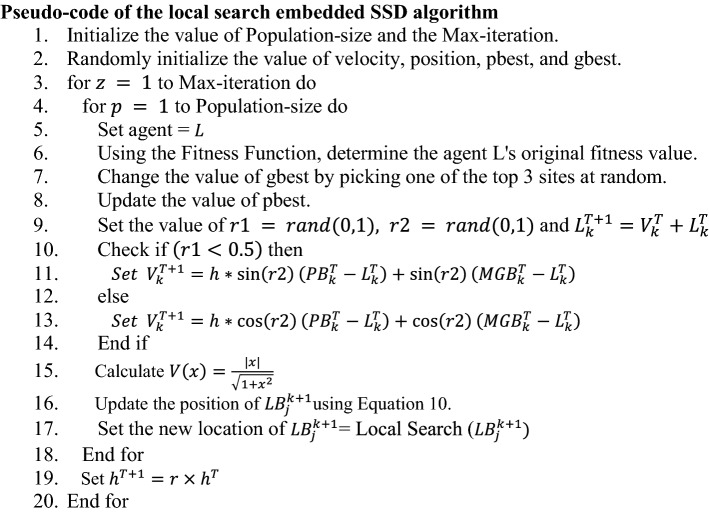


### Fitness function

The motive of this particular section is to describe how to evaluate the quality of a candidate solution. A learning algorithm needs to be exploited for assessing as SSD is a wrapper-based algorithm. Hence, we have exploited the KNN [68] classifier for the computation of classification accuracy of a candidate solution, succeeding the works of [69–71]. The fitness function mainly contains two components: one is the number of features and another one is classification accuracy. These components are contradictory to each other. We have to increase classification accuracy but at the same time, we need to decrease the number of features selected. So we have determined to exploit the classification error. As a lesser error value would indicate a better fitness score, so would a lesser number of features. In Eq. (11), the fitness function used to assess a given feature set’s strength is defined.

11 $$ Fitness\;Function = w \times \propto + \left( {1 - w} \right) \times \frac{\left| s \right|}{{\left| d \right|}} $$

In Eq. (11), $|d|$ is the total count of features in the dataset, $|s|$ denotes the count of features in the chosen feature set (i.e., a candidate solution),$\propto$ is the error in classification using the feature subset, and $w\in [0, 1]$ signifies the relative weight value given to the classification error and the number of features.

## Experimental results and discussion

In this section, we describe the dataset used in the current study and report the results obtained by applying our proposed method on the dataset. To justify the superiority of the framework, comparisons to other published methods on the same dataset have also been discussed.

### Experimental setup

We perform all the experiments on a machine with 12 GB NVIDIA Tesla T4 GPU and the programming language used is Python 3.6. The deep learning model is implemented with the Tensorflow environment using the Keras library.

#### Dataset description

**Fig. 5:**
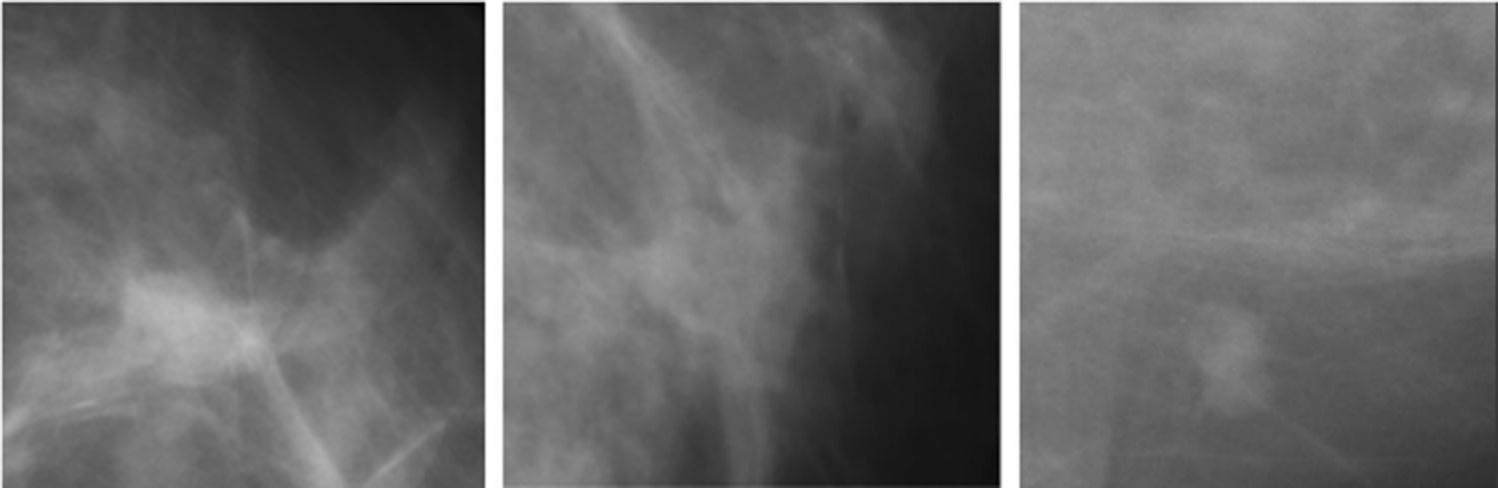
Sample images of Benign breast mass from the DDSM dataset

**Fig. 6:**
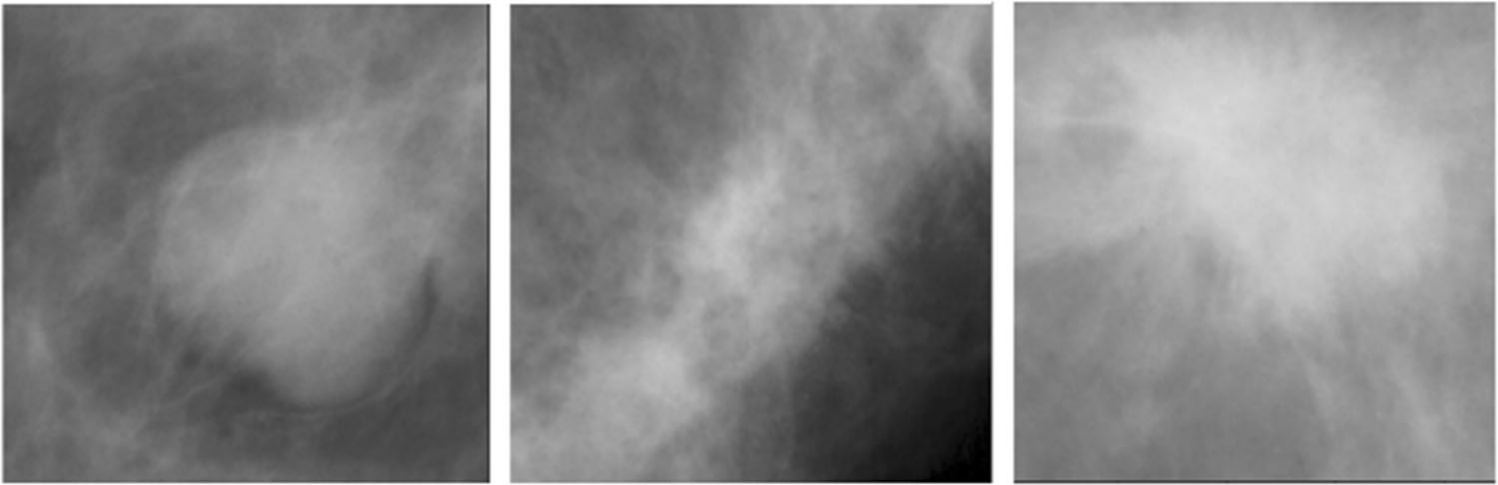
Sample images of Malignant breast mass from the DDSM dataset

We assess our model on a publicly accessible breast cancer mammography database [72] which is an open-source and unbalanced binary mammography image dataset that contains normal images i.e., negative samples from the DDSM database [73], and malignant images i.e., positive samples from the CBIS-DDSM database [74]. It has a total of 55,885 training samples out of which 86% are negative samples and 14% are positive samples. The dimension of each sample is 299 × 299. In this work, we consider only the training samples of mammogram masses which are categorized as benign and malignant masses. Sample images of benign and malignant masses of the DDSM dataset are shown in Figs. 5 and 6, respectively.

#### Performance evaluation metrics

We assess our model with the following evaluation metrics:

The ratio of accurately predicted labels to the overall size of the dataset is referred to as accuracy. [75]. Accuracy is calculated as follows:

12 $$Accuracy = \frac{TP + TN}{{\left( {TP + TN + FP + FN} \right)}}$$

The percentage of samples identified as positive that are actually positive is known as Precision. It is the ratio of successfully predicted positive class labels to the total number of positive class samples predicted [75]. It is calculated as follows:

13 $$Precision = \frac{TP}{{\left( {TP + FP} \right)}}$$

Recall is calculated by dividing the number of true positive samples by the total number of positive samples in that class. [75].

14 $$Recall = \frac{TP}{{\left( {TP + FN} \right)}}$$

where True Positive (TP) indicates the positive class samples that the classifier correctly labels, True Negative (TN) indicates the negative class samples that the classifier correctly labels, False Positive (FP) indicates the negative class samples that were incorrectly identified as positive class samples, and positive class samples that have been mislabeled as negative class samples are known as False Negatives (FN).

#### Parameter tuning for transfer learning

**Fig. 7:**
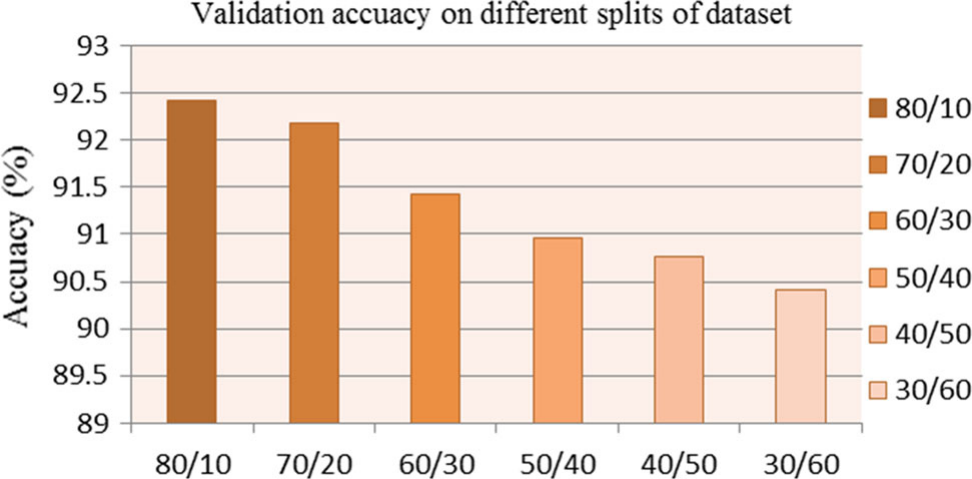
The attention-aided VGG16 model’s validation accuracy for varied splits of training and validation data. On the DDSM database, the model gets the best classification accuracy for 80% training and 10% validation data

The experiments are carried out and assessed on the aforementioned database, which contains 80% training data, 10% testing data, and 10% validation data. We started by experimenting with alternative training and validation data splits. Figure 7 shows the experimental results from the DDSM database. For the above-mentioned splitting, we find that the model has the maximum classification accuracy. In practice, we experiment with various typical parameters for learning rate and batch size to find the best possible combination. We take into account the initial learning rate $\in${1e-2, 1e-3, 1e-4, 1e-5, 1e-6} and batch size $\in${8, 16, 32, 64} and come up with a decent combination of 1e-2 and 64 as the initial learning rate and batch size. In addition, for training purposes, we employ the popularly used Adam optimizer. We employ a step learning rate scheduler for smooth learning, where the learning rate is lowered by a factor of two after the third epoch. The dropout values for the two dropout layers are 0.5 and 0.25, respectively. Table 1 shows the exact parameter values for training the model necessary to perform our strategy.

**Table 1:** Parameter details used in this work for the training of the DL based transfer learning models

Parameter	Value
Batch size	64
Optimizer	Adam
Initial learning rate	0.001
Loss function	BCE

#### Parameter tuning for SSD-based FS

In this subsection, we discuss the range of values of various parameters and the fitness function used in the SSD algorithm. We want to reduce our fitness function specified in Eq. (11), i.e., the number of features as well as the classification error, as a critical element of this task. If we increase the value of $w$ in the fitness function then we give more importance to deducting the number of features, whereas if we assign a minimum value to $w$ then more emphasis is given to reduce the classification error. A series of experiments with different values of $w$ leads to the optimal value of $w$ being 0.2. As a result, the algorithm under consideration prioritizes minimizing the classification error, i.e., increasing the classification accuracy. We have tested our strategy using a variety of $h$ and $r$ values in the search space throughout the experimentation. Our investigations also show that as the value of $h$ is raised, the accuracy of the classification gets improved. When the value of $r$ is lowered, the classification performance improves but eventually declines after reaching a peak. When the value of $r$ is too low, this results in overfitting. When the values of $h$ and $r$ are fixed at 100 and 0.9, respectively, the maximum classification accuracy is reached.

### Performance of attention-aided deep feature extraction model

As mentioned earlier, in this work, at first we consider a deep attention model for the extraction of deep features from the mammogram inputs. We use the VGG16 model as our base model and incorporate an attention method to this. We experiment with some popular pre-trained end-to-end models for deep feature extraction and report the result in Table 2. Furthermore, we incorporate the attention mechanism with each of these models. Noteworthy improvement in classification accuracy is achieved due to the effect of weighted average pooling attention. As in the last layer of the CNN.

**Table 2:** Performance of different pre-trained transfer learning (TL) models on the DDSM database

Pre-trained TL model	Accuracy (%)
VGG19	87.45
ResNet50	87.23
EfficientNet	87.22
VGG16	87.30

model as an alternative to the fully connected layer, we consider using the GAP layer and it gives a single feature map for the corresponding category. This layer uses spatial information by enforcing correspondences between feature maps and categories. The results obtained on the test dataset are tabulated in Table 3. The Mean and the standard deviation (SD) value are shown over five simulations of the obtained results. From Table 3, it can be observed that the VGG16 model with attention classifies the mammograms more appropriately. Hence, we decide to proceed with the attention-based VGG16 model.

**Table 3:** Performance of the attention-aided different deep feature extraction models on the DDSM database

Attention-aided TL model’s accuracy (%)				
Simulation	VGG16	VGG19	ResNet50	EfficientNet
1	92.42	91.89	89.96	89.89
2	91.86	91.94	91.14	91.17
3	92.12	91.77	91.32	90.78
4	91.41	90.34	91.23	89.89
5	91.51	90.78	91.86	90.34
Mean ± SD	91.86 ± 0.42	91.34 ± 0.73	91.10 ± 0.69	90.41 ± 0.56

**Fig. 8:**
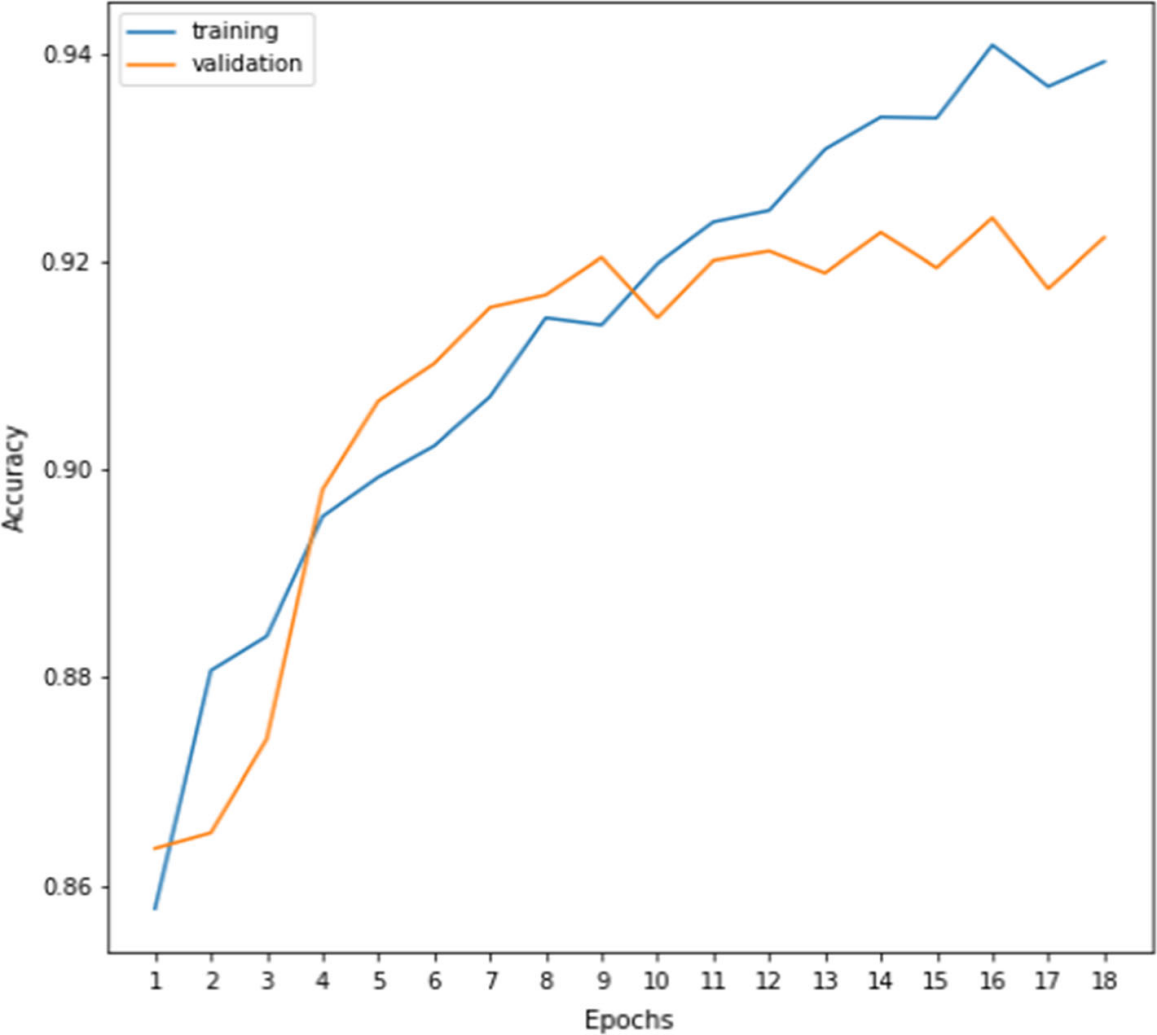
Training and validation accuracies (%) over the number of epochs of the attention-aided VGG16 model on the DDSM database

**Fig. 9:**
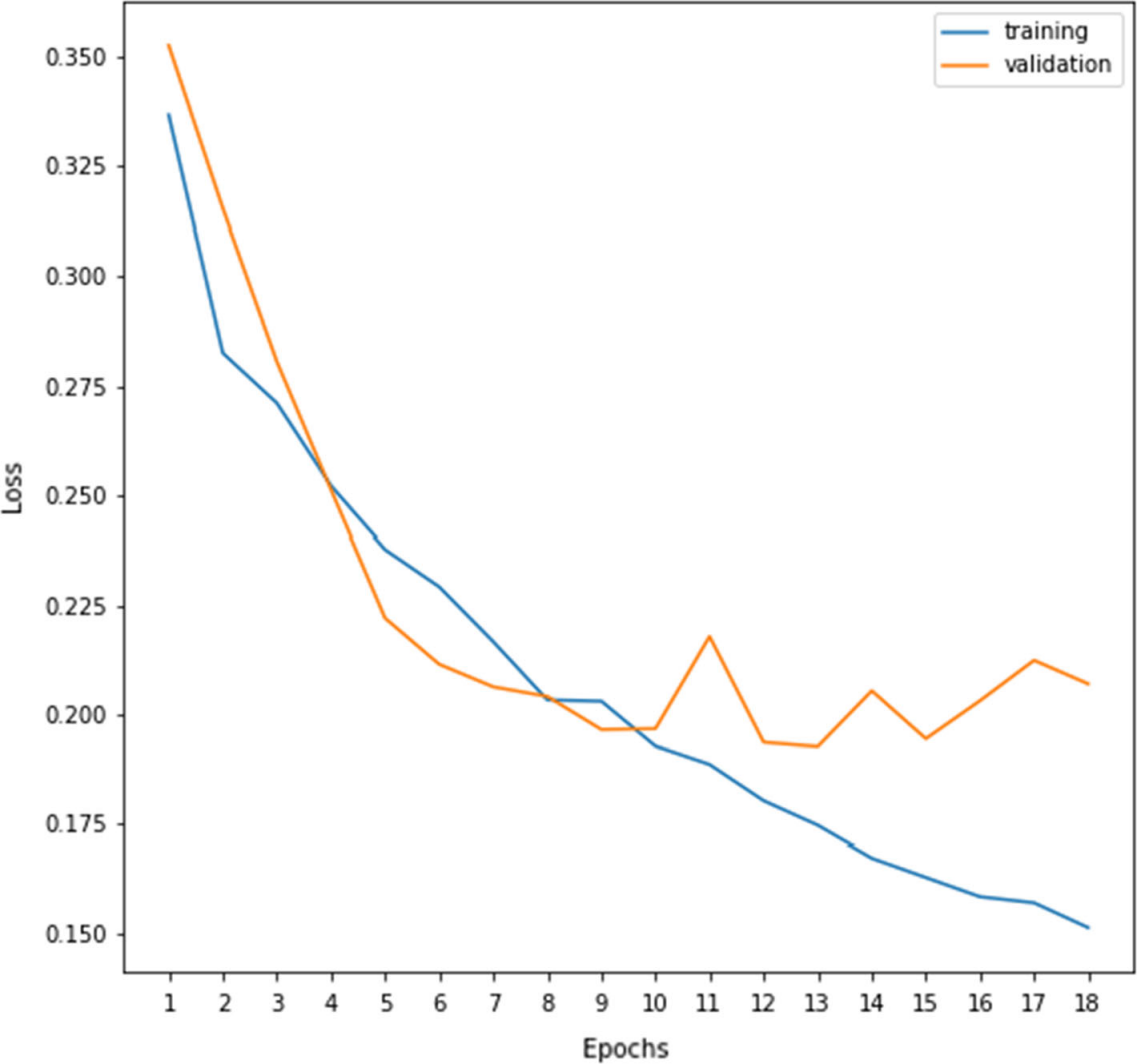
Training and validation loss values (%) over the number of epochs of the attention-aided VGG16 model on the DDSM database

During the training of the attention-aided VGG16 model, training and validation accuracies for every epoch of the first simulation are recorded and shown in Fig. 8. It can be seen from Fig. 8 that initially the model does not suffer from any major over-fitting and later on the validation accuracy does not improve much and the accuracy oscillates between the values 91 to 92, whereas Fig. 9 displays smooth learning of the model during training. It must be noted that all the values in Fig. 9 are evaluated using the widely adopted binary cross-entropy (BCE) loss function. Deep features extracted from the VGG16 model are fed to different local-search embedded FS algorithms and the results are discussed in the subsequent sections.

### Performance of the local search embedded SSD algorithm

We apply a local search-based FS algorithm to gain the maximum possible accuracy as well as to reduce the number of features used for the classification purpose. As finding the best feature subset is a difficult task, especially in a wrapper-based FS model, we combine a local search method with an FS model. The results obtained from the proposed method are shown in Table 4. Table 4 shows that high accuracy of 96.07%, the precision of 96.30%, sensitivity (recall) of 99.28% and feature dimension reduction of 75% have been achieved.

**Table 4:** Performance of the proposed method on the test set of DDSM database over five simulations

Simulation	Accuracy (%)	Precision (%)	Recall (%)	#FS
1	96.43	96.97	98.97	32
2	95.98	95.57	100.0	30
3	96.43	96.97	98.97	35
4	95.54	95.54	99.48	34
5	95.98	96.48	98.97	31
Mean ± SD	96.07 ± 0.37	96.30 ± 0.71	99.28 ± 0.21	32 ± 2

For experimentation, we use two well-known local search methods, namely LAHC [76] and ABHC [64] to optimize the feature set. The experimental result on the DDSM database with the said local search-embedded SSD FS method is shown in Table 5. From Table 5, it is clear that the ABHC embedded SSD technique outperforms the LAHC embedded method in terms of classification accuracy as well as obtaining a smaller optimal feature set.

**Table 5:** Performance of the ABHC and LAHC local search embedded SSD algorithm on extracted deep features from the VGG16 model. Classification accuracy is in %

Simulation	Local search embedded SSD			
	SSD + LAHC		SSD + ABHC	
	Classification accuracy	No. of selected features	Classification accuracy	No. of selected features
1	94.19	52	96.43	32
2	94.64	59	95.98	30
3	94.28	53	96.43	35
4	93.98	56	95.54	34
5	93.86	47	95.98	31
Mean ± SD	94.19 ± 0.30	53 ± 5	96.07 ± 0.37	32 ± 2

### Comparison with various meta-heuristic based FS methods

We perform experiments using various other meta-heuristic based FS methods namely: Gravitational Search Algorithm (GSA) [77], WOA [78], Gray-wolf Optimization (GWO) [79], GA [80], PSO [81], Sine Cosine Algorithm (SCA) [82], Harmony Search (HS) algorithm [83] and Equilibrium Optimizer (EO) [84] for comparison of the proposed method. Meta-heuristic-based FS algorithms need to perform many mathematical operations to identify the best feature subset. For this, the algorithms in the literature use many sets of equations that are aided by different parameters. These parameters are crucial for controlling the optimization process and they have their own significance. The standard values of these parameters of various algorithms are used in this study. Table 6 contains a list of the parameters and their values. The simulation results of local search-based different meta-heuristics FS algorithms are shown in Table 7.

**Table 6:** Different sets of hyperparameters and their values for various meta-heuristic based FS algorithms considered for the experimentation purposes

FS algorithm	Parameter(s)	Value(s)
Generic parameters	Population size	20
	Number of iterations	100
	Weight for accuracy (α	*α* = 0.98
GSA	Initial gravitational constant (G_init_)	G_init_ = 6
	Constant (*ε*)	0.00001
WOA	Encircling parameter (a)	a lies in [0 2]
	Shape of spiral (b)	b = 1
GWO	Convergence operator (a)	a lies in [0 2]
GA	Gene selection	Roulette wheel
	Crossover probability	0.4
	Mutation probability	0.3
PSO	Inertia weight (I_W_)	I_W_ lies in [0 1]
	Coefficients (r_1_, r_2_)	r_1_ and r_2_ lies in [0 1]
SCA	Constant (a)	a = 3
	Movement direction (r_1_)	r_1_ lies in [0 3]
HS	Harmony memory	HMCR = 0.90
	Considering rate (HMCR)	
EO	Pool size	4
	Constants (a_1_, a_2_)	a_1_ = 2 and a_2_ = 1
	Generation rate (GP)	GP = 0.5

**Table 7:** Results from five simulations, including average and standard deviation (SD), after deep features from the VGG16 model are extracted and fed to several FS algorithms with embedded local search

FS method	LAHC embedded FS method		ABHC embedded FS method	
	Classification accuracy (%)	No. of features selected	Classification accuracy (%)	No. of features selected
GSA	93.30	65	95.08	70
	94.28	54	94.19	63
	94.19	51	94.28	56
	93.30	67	92.41	57
	93.33	65	93.30	64
Mean ± SD	93.68 ± 0.51	54 ± 7	93.85 ± 0.92	62 ± 6
WOA	94.64	48	94.64	55
	93.33	58	93.75	58
	93.75	59	93.33	61
	94.19	68	94.64	69
	94.19	48	94.64	60
Mean ± SD	94.02 ± 0.50	56 ± 8	94.19 ± 0.55	61 ± 5
GWO	94.64	76	94.64	69
	93.33	65	93.30	78
	93.75	81	93.33	63
	94.64	74	93.75	79
	93.33	65	94.64	75
Mean ± SD	93.94 ± 0.67	72 ± 7	93.93 ± 0.59	73 ± 7
GA	94.64	70	94.64	67
	93.30	59	93.75	62
	93.30	57	93.75	55
	93.33	56	93.33	59
	94.64	70	93.30	59
Mean ± SD	93.84 ± 0.73	62 ± 7	93.75 ± 0.48	60 ± 4
PSO	93.30	41	94.64	55
	93.33	42	94.19	52
	94.19	50	93.30	51
	92.86	46	93.33	48
	93.33	52	94.64	57
Mean ± SD	93.40 ± 0.48	46 ± 5	94.02 ± 0.59	53 ± 4
SCA	93.30	59	94.19	70
	93.33	71	93.75	67
	94.19	73	93.75	64
	93.33	65	93.33	63
	93.41	57	94.19	70
Mean ± SD	93.51 ± 0.38	65 ± 7	93.84 ± 0.32	67 ± 3
HS	93.30	63	92.41	60
	94.28	63	91.16	73
	95.08	56	93.33	53
	93.33	55	91.52	62
	92.42	65	91.96	68
Mean ± SD	93.68 ± 1.02	60 ± 5	92.07 ± 0.75	63 ± 8
EO	93.75	53	94.64	55
	94.28	56	94.64	69
	94.64	56	93.30	55
	94.64	50	94.28	63
	93.41	55	93.33	55
Mean ± SD	94.14 ± 0.55	54 ± 3	94.04 ± 0.60	59 ± 6

**Fig. 10:**
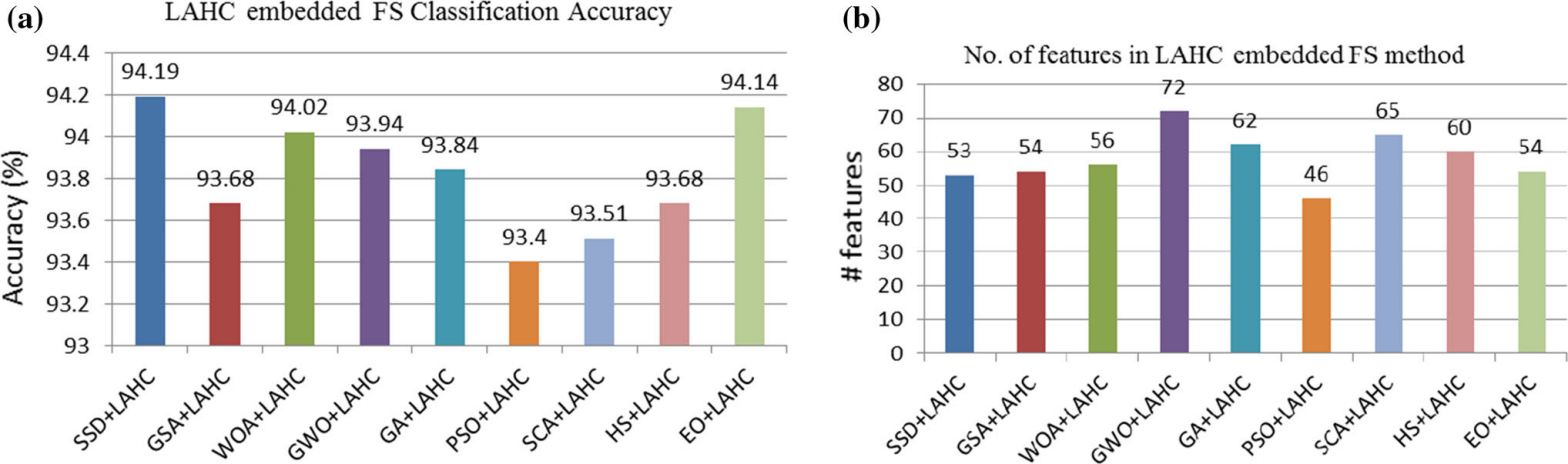
Results of different FS algorithms embedded with LAHC local search

**Fig. 11:**
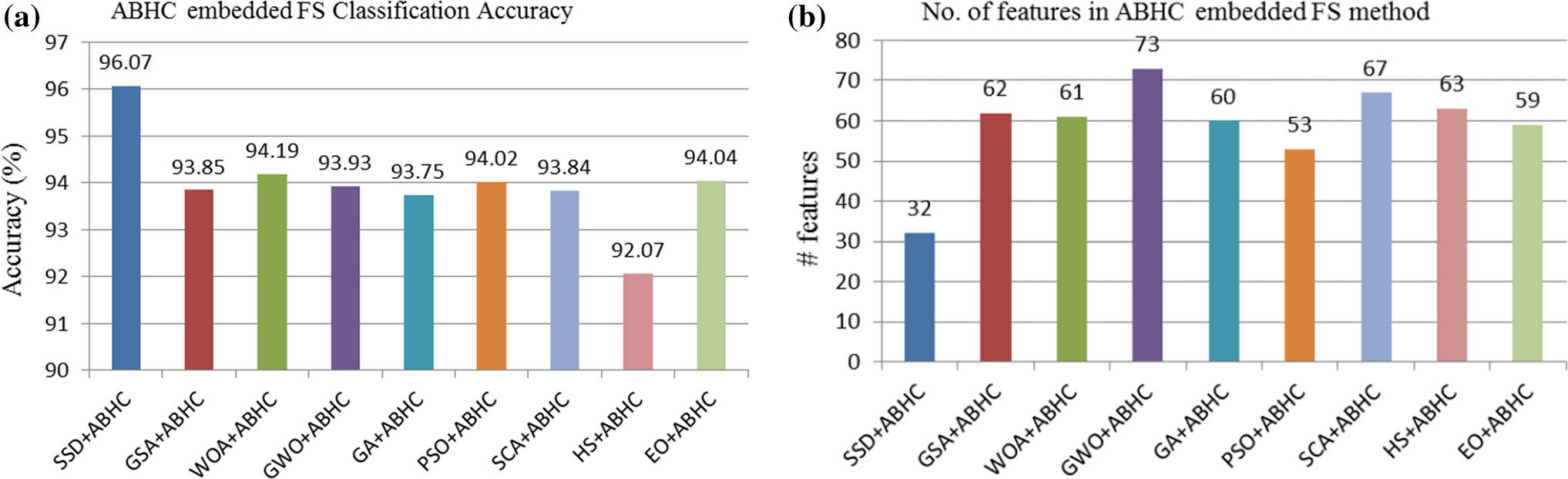
Results of different FS algorithms embedded with ABHC local search (a) Classification accuracy (b) No. of selected features

From Tables 5 and 7, we can say that ABHC local search-embedded SSD algorithm outperforms others in terms of classification accuracy. Besides, it provides a subset of 32 features which is just 25% features of the given input features obtained from the VGG16 model. The comparative analysis with different combinations of various meta-heuristics and local search is shown in Figs. 10–11. The sine and cosine functions complicate the movement direction of the agents, which is the most essential feature of this SSD-based FS technique. This allows the algorithm to diversify, and the parameter $h$ in Eq. (3) ensures that the algorithm remains stable between exploration and exploitation, allowing it to converge to better solutions. Furthermore, ABHC aids the algorithm to improve the solutions, thereby overcoming the local optima, thus leading to a better outcome.

Besides, ABHC based SSD method achieves not only better classification accuracy but also yields high precision and recall values which is crucial for medical image analysis. However, LAHC and ABHC, both the local search methods are based on the hill-climbing optimization technique. The methods differ in the way of finding the better agent having better fitness value toward the final reduced solution.

**Fig. 12:**
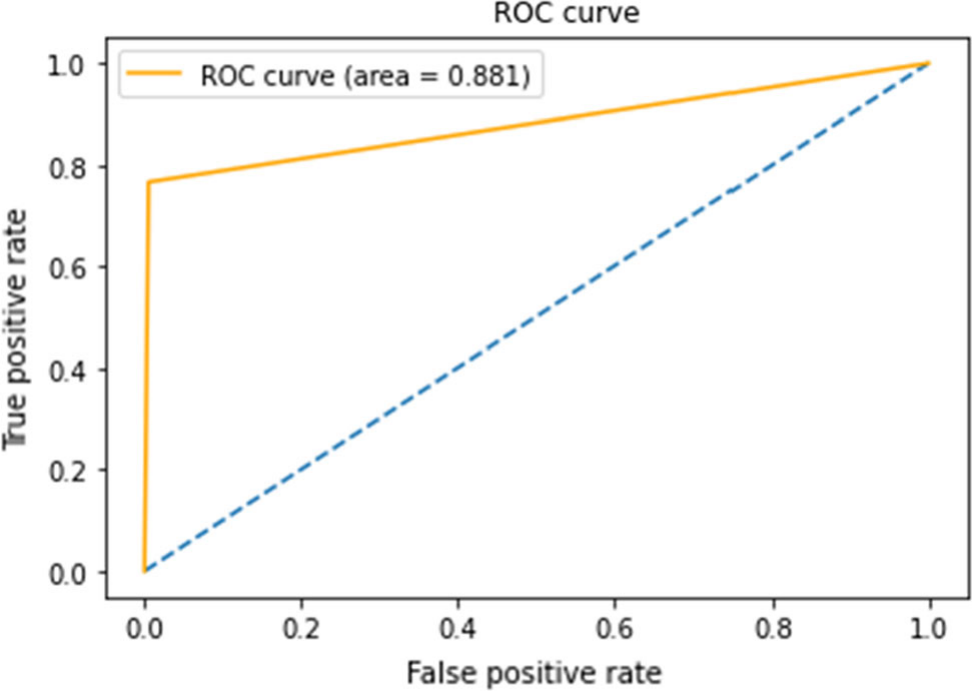
The ROC curve with the AUC value of the DDSM database using our proposed method

Figure 12 depicts the suggested method’s Receiver Operating Characteristic (ROC) curve, which has an AUC value of 0.881. The ROC curves depict the trade-off between a classifier’s true positive rate (TPR) and false positive rate (FPR). Classifiers with curves that are closer to the top-left corner perform better. If the curve approaches the ROC space’s 45-degree diagonal, the classification result becomes less accurate.

Besides, from Fig. 11, it can be said that our proposed model yields a promising result not only in terms of the classification accuracy but also the same is achieved with a very less number of features that we get from the ABHC embedded SSD FS algorithm as compared to other FS algorithms.

Here the FS algorithm produces the optimal subset of features by discarding redundant or less informative features from the deep features obtained from the CNN model and local search increases the exploitation capability of the FS algorithm by finding the location of an agent in the search space having better fitness value.

Furthermore, we experiment with different modules of our proposed method to observe the effectiveness of each module and noted the experimental results in Table 8. From the table, it can be observed that an attention-aided VGG16 model yields a classification accuracy of 91.41% on the test set, whereas if we use the KNN classifier to it, the classifier produces a classification accuracy of 90.70% which is low compared to end-to-end VGG16 model. Also, if we apply FS using the SSD algorithm, improved classification accuracy is observed. Further, if the ABHC local search is incorporated with the SSD-based FS method, we achieve improved classification accuracy. Therefore, from these experimental results, it can be ensured the effectiveness of the individual modules (i.e., deep feature extraction with VGG16, FS with ABHC aided SSD method) of our proposed method. The gain in performance has to be attributed to both the DL and FS approaches.

**Table 8:** Comparative results for different combinations of the proposed model in terms of classification accuracy (%) on the test set of the DDSM database

Method	Classification accuracy (%)
End-to-end VGG16	91.41
VGG16 + KNN	90.70
VGG16 + SSD + KNN	92.86
VGG16 + ABHC aided SSD + KNN	95.98

### Computational complexity analysis of the proposed method

The asymptotic analysis is performed on the proposed ABHC embedded SSD method. The space complexity of the proposed method is $Big-O (PS * Dim)$, where $PS$ is the population size and $Dim$ is the feature dimension. The time complexity of the ABHC-embedded SSD method shows that the worst-case time cost in asymptotic notation is

$$ Big - O\left( {Max\;\;iteration \times PS \times \left( {T_{{fit}}  + T_{{knn}}  + Dim} \right)} \right). $$

where the Maxiteration is the maximum number of iterations for the local search-based FS method, ${T}_{fit}$ is the time to calculate fitness function and ${T}_{knn}$ is the time to run KNN algorithm.

### Statistical analysis of the proposed method

We perform a statistical significance test to assess the proposed algorithm’s robustness in comparison to other meta-heuristic algorithms embedded with the ABHC-based local search. The following statement is taken into account as a null hypothesis while we do this test: “The proposed ABHC local search aided SSD method gives similar outcomes when compared to other meta-heuristic techniques embedded with the ABHC based local search.” We use the Mann–Whitney U test [91], a widely used nonparametric statistical technique, to reject this null hypothesis. This test is predicated on the notion that two distributions, *X* and *Y*, are ranked in ascending order according to their respective values. The majority of the samples in *X* must be above or below the majority of the samples in *Y* for a condition to hold [91]. We take into account the classification accuracy of different FS techniques for each of the five simulations to construct the statistical evidence. The results obtained from performing the test are shown in Table 9. If the calculated *p*-value is higher than 0.05 (5%), we conclude that the null hypothesis has sufficient statistical support to be accepted. If not, we reject this hypothesis. It can be seen from Table 9 that for every case *p*-value is less than 0.05 which means that the ABHC local search aided SSD method is statistically different from the other methods considered here.

**Table 9:** Results obtained on statistically analyzing the ABHC aided SSD algorithm with other FS algorithms embedded with the ABHC based local search using Mann–Whitney U test

ABHC embedded FS method	*p*-value
ABHC + GSA	0.01167
ABHC + WOA	0.01066
ABHC + GWO	0.01141
ABHC + GA	0.01192
ABHC + PSO	0.01141
ABHC + SCA	0.01115
ABHC + HS	0.01218
ABHC + EO	0.01208

### Comparison with state-of-the-art approaches

Finally, we compare the performance of the proposed method with some recently developed classification methods and tabulate the same in Table 10. The performance of our method is superior to that of four DL-based methods used in [4, 41, 85], and [86]. Moreover, the performances of the method described in [87, 88], based on feature fusion to classify benign and malignant masses, are slightly lower than that of our method. Hence, from Table 10, it is clear that the proposed model outperforms these previous works with a good margin and the experimental results establish the superiority and robustness of our proposed method.

**Table 10:** Performance comparison of the proposed model with state-of-the-art models on the DDSM database

Model	Dataset	Accuracy (%)	Precision (%)	Recall (%)
Levy et al. [4]	DDSM	92.9	92.4	93.4
Falconi et al. [41]		84.4	–	–
Xiao et al. [85]		–	82.2	94.9
Arias et al. [86]		92	–	–
Zhang et al. [87]		94.30	–	89.97
Li et al. [88]		94.7	–	94.1
Proposed		96.07	96.30	99.28

### Advantages and limitations of the proposed method

Although our proposed method poses good results for cancer detection in mamograms, there are some pros and cons to this work. In this section, we discuss the advantages and limitations of our proposed method. The core advantages of this current research work are as follows:

1. We create a model for breast cancer classification from mammograms that combines the principles of deep learning and optimization algorithms.
2. We introduce an attention mechanism on a deep CNN-based transfer learning model, called VGG-16, and fine-tune it for the extraction of deep features from the input images.
3. We embed a local search, namely ABHC with SSD based FS algorithm to produce an optimal feature subset from the features produced by the said CNN model.
4. We achieve state-of-the-art classification accuracy, high precision and recall values with just 25% of features of the original feature set obtained by the CNN model when evaluated on the DDSM database.

The limitations of this research work are described as follows:

1. The initialization in the optimization algorithm is random. So it may sacrifice some results in terms of accuracy and convergence time. Instead of random initialization, techniques like a chaotic map can be explored for better results.
2. Time complexity is a factor on which we need to put more emphasis in the future.
3. Another issue is it may result in early convergence for some inputs.

## Conclusions and future works

Breast cancer is a significant problem that affects women all over the world, therefore it is critical to recognize any early signs of the disease and treat it with the help of medical specialists. We present a model for breast cancer categorization based on mammography masses in this paper. First, we extract features from the VGG16 model with care. We use the GAP layer instead of the fully connected layer to implement an attention mechanism over the original VGG16 model. Because it enforces the relationship between a feature map and the category, it is more organic to the convolution construction. We then fed the retrieved features into a local search embedded FS model, and it was discovered that the ABHC embedded SSD technique outperforms the local search embedded FS model with a smaller number of features. The FS algorithm minimizes the quantity of features, but local search improves the FS method’s exploitation potential and yields the best subset of features. With just 25% of features extracted by the DL model, our proposed model achieves state-of-the-art classification accuracy, precision, and recall on the DDSM dataset. Other medical image datasets of other modalities can be examined in the future to verify the resilience of the proposed model, as the notion of the present study is dataset independent. Also, the present work deals with a classification problem, whereas the segmentation of breast lesions is another challenging area in the medical image analysis that can be explored in future research attempts. Furthermore, the SSD algorithm’s fitness function and position updating approach are essential variables for feature reduction that may be enhanced further. Also, parallel methods can be used to speed up computations on higher-dimensional datasets.

## Data Availability

The dataset used and analyzed during the current study is publicly available at: https://www.kaggle.com/datasets/skooch/ddsm-mammography.
